# Considerations on inhibition of IL‐23 in psoriatic women of childbearing potential

**DOI:** 10.1111/dth.14931

**Published:** 2021-03-17

**Authors:** Roberto Russo, Giulia Gasparini, Emanuele Cozzani, Martina Burlando, Aurora Parodi

**Affiliations:** ^1^ Di.S.Sal. Section of Dermatology University of Genoa Genoa Italy; ^2^ IRCCS San Martino Polyclinic Hospital Genoa Italy; ^3^ Department of Experimental Medicine (DIMES) University of Genoa Genoa Italy

**Keywords:** biologic drugs, biologics, pregnancy, psoriasis

## Abstract

Lack of safety data on pregnant women determines difficulty in choosing the correct biologic agent to treat psoriasis in women of childbearing potential. Studies have postulated a role of IL‐23 in unexplained recurrent spontaneous abortions. This gives rise to consideration about use of anti‐IL‐23 drugs in treatment of psoriasis in women of childbearing potential.

IL‐23 is an important cytokine involved in the pathogenesis of psoriasis; biologic agents directed towards IL‐23 represent new options for treatment of psoriasis.^1^ To date, there are not certain data on safety of biologic drugs in the treatment of pregnant women, although studies have been published. Certolizumab seems to have the best safety profile on pregnant women, as its placental transfer is nonexistent to minimal.^2^ A real‐life, multicenter retrospective analysis reported only one spontaneous abortion among 14 pregnancies occurred during treatment with biologics for psoriasis.^3^ A systematic review showed increased risk of congenital malformation and preterm birth among women treated with Tumor Necrosis Factor‐α inhibitors.^4^

Table 1 summarizes data on pregnancies occurred during clinical trials of anti‐IL‐23 agents for psoriasis.

**TABLE 1:** Pregnancies occurred in psoriatic women receiving at least one dose of anti‐IL‐23 agents during clinical trials

	*n* of pregnancies	Spontaneous abortions	Premature births
Guselkumab	7	0	0
Tildrakizumab	14	2	1
Risankizumab	n/a	n/a	n/a

A post hoc analysis of data from tildrakizumab clinical trials for psoriasis revealed a total of 14 pregnancies among 528 female patients treated. In all cases, treatment was discontinued after confirmation of pregnancy. Four pregnancies resulted in elective abortion. Seven pregnancies resulted in full‐term births, newborns had no anomalies except one with transient jaundice. One premature birth (36 weeks) occurred, without anomalies in the newborn. Spontaneous abortions occurred in two cases, at 4 and 8 weeks respectively. The rate of spontaneous abortions (14%) was similar to that of general population (12‐15%). Of note, the patients whose pregnancies resulted in premature birth or spontaneous abortions did not receive any dose of tildrakizumab during pregnancy. Three patients whose pregnancies resulted in full‐term births were at least 35 years old.^5^

Data from pregnancies during guselkumab clinical trials showed no premature births; two spontaneous abortions were reported, involving women enrolled as healthy controls. Seven patients receiving guselkumab for psoriasis had pregnancies resulting in full‐term births.^6^

No data on pregnant patients could be retrieved from clinical trials for risankizumab in psoriasis (IMMvent, UltIMMa‐1 and ‐2, IMMhance).^7^

It has been proposed that IL‐23 may play a role in unexplained recurrent spontaneous abortions (URSA), defined as at least two consecutive abortions before week 12 of gestation, excluded any verifiable causes. In fact, increased levels of IL‐23, as well as of IL‐17, IL‐17R, and p‐STAT3, were found in peripheral blood and in deciduae of women with unexplained recurrent spontaneous abortions in their first trimester of pregnancy, compared to women in normal early pregnancies.^8^

A study on decidual samples from 15 healthy pregnant women demonstrated that treatment of decidual immune cells with IL‐23 resulted in unbalance of Th17/Treg ratio in favor of Th17 and in hypersecretion of IL‐1B and IL‐17; oppositely, treatment with anti‐IL‐23 antibody significantly increased the Th17 percentage and decreased the production of IL‐1B and IL‐17, whereas it amplified the secretion of IL‐10. Moreover, the expression of p‐STAT3 was significantly elevated by treatment with IL‐23 and inhibited by treatment with anti‐IL‐23 antibody.^9^ Considering the role of IL‐23 in differentiation and expansion of Th17 cells,^10^ these findings are of interest. In fact, Th17/Treg ratios are important to successful pregnancy, so they are regarded as therapeutic targets for URSA.^11^, ^12^, ^13^ Specifically, remarkably higher amounts of Th17 cells have been found in the peripheral blood and decidua of women with URSA,^11^, ^14^, ^15^ whereas normal pregnancy is characterized by Th2 and Treg dominance.^16^ Moreover, higher levels of IL‐1B and IL‐17 have been found in women with URSA.^17^

In our experience of 650 psoriatic patients treated with biologics in our Clinic, four pregnancies occurred: of them, two patients were on adalimumab, one on ustekinumab, one on guselkumab. All patients discontinued their treatment as soon as pregnancies were discovered. No abortion, no premature birth occurred; all newborns had no anomalies. Of course, our data are still insufficient to prove anything.

Undeniably, the actual lack of certain data urges to advise use of contraception during biologic treatment for psoriasis, as well as drug withdrawal if pregnancy occurs. Anyway, considering the available data, anti‐IL‐23 agents do not seem to affect pregnancy outcomes in patients with psoriasis.

Provided that their administration is discontinued as soon as gestation is discovered, they seem to be a quite safe option for psoriasis in women of childbearing potential.

## CONFLICT OF INTEREST

The authors declare no conflict of interest.

## AUTHOR CONTRIBUTIONS

All the authors listed contributed to data search and analysis and to the writing and revising of the manuscript.

## Data Availability

The data that support the findings of this study are available in the studies cited in the reference list.
